# Formulation of Topical Drug Delivery Systems Containing a Fixed-Dose Isoniazid–Rifampicin Combination Using the Self-Emulsification Mechanism †

**DOI:** 10.3390/pharmaceutics17060680

**Published:** 2025-05-22

**Authors:** Melissa van Deventer, Richard K. Haynes, Marius Brits, Joe M. Viljoen

**Affiliations:** 1 Centre of Excellence for Pharmaceutical Sciences (Pharmacen^TM^), Building G16, Faculty of Health Sciences, North-West University, Potchefstroom 2520, South Africa; melissa.vd1803@gmail.com (M.v.D.); rihaynes@csu.edu.au (R.K.H.); marius.brits@nwu.ac.za (M.B.); 2 Rural Health Research Institute, Charles Sturt University, Orange, NSW 2800, Australia

**Keywords:** cutaneous tuberculosis (CTB), isoniazid (INH), rifampicin (RIF), self-emulsifying mechanism, topical delivery, pseudoternary phase diagrams

## Abstract

**Background**: Tuberculosis remains a significant global health issue, and the rise of drug-resistant strains is becoming increasingly concerning. Currently, treatment options are limited to systemic regimens; however, developing topical drug delivery systems could offer advantages for treating cutaneous tuberculosis (CTB) when applied directly to the lesions. We developed topical emulsions using the self-emulsification mechanism that combine fixed doses of isoniazid (INH) and rifampicin (RIF) using a quality-by-design approach. **Methods**: Preformulation studies pertaining to drug solubility in various solvents, the construction of pseudoternary diagrams to identify self-emulsification regions for each tested excipient combination, and the preparation of checkpoint formulations were conducted and visually examined. Formulations displaying no physical instabilities were subsequently exposed to characterization experiments, including droplet size determination, zeta potential, size distribution, viscosity, pH, self-emulsification, cloud point, robustness to dilution, and thermodynamic stability assessment. Three selected formulations were consequently subjected to membrane release experiments, followed by skin diffusion studies, and INH and RIF stability in these emulsions was determined, because these drugs have a known interaction. **Conclusions**: While incorporating essential oils in a topical formulation improved RIF solubility, it also resulted in several instabilities. RIF exhibited greater susceptibility to degradation under higher temperatures and lower pH conditions. However, drug release from all formulations tested was confirmed. Notably, olive oil microemulsions demonstrated the most favorable characteristics for dermal drug delivery; nonetheless, drug diffusion into and through the skin (which was not desired) could not be quantified. Despite these challenges, the findings indicate that topical drug delivery systems using the self-emulsification process can facilitate the direct treatment of CTB.

## 1. Introduction

The effective treatment of tuberculosis (TB), including cutaneous TB (CTB), faces many challenges, including drug interactions due to severe adverse effects, poor patient adherence, increased drug resistance, and lengthy treatment regimens [1,2,3]. Furthermore, drugs used for TB treatment often have poor pharmacokinetic properties, such as low absorption and bioavailability [4,5,6]. These aspects emphasize the overwhelming need for the development of new drug entities coupled with the utilization of new or modified drug delivery systems that improve pharmacokinetic properties and thereby enhance the efficacy of prescribed therapy.

In recent years, the use of fixed-dose combinations (FDCs) for TB treatment has become increasingly popular to decrease the large tablet count associated with TB therapy and to combat the increasing drug resistance of TB bacterial strains [7,8]. Rifampicin (RIF), one of the frontline drugs used against TB, is often combined with isoniazid (INH), ethambutol, and pyrazinamide in established FDCs [9]. However, this drug is thermally relatively unstable and undergoes increased degradation when combined with INH and/or is exposed to an acidic environment such as the stomach [10,11]. Interestingly, the inclusion of ethambutol and pyrazinamide together with INH and RIF in an FDC, which also forms part of the first-line treatment regimen, has been shown to enhance degradation. Moreover, RIF also interacts with other drugs that may be co-administered in an attempt to address resistant TB [4]. Therefore, attention should be given to developing dosage forms that maintain the physical separation of INH and RIF in an FDC to improve the stability and subsequent bioavailability of RIF [11].

Lately, micro- and nanocarriers have been examined for the delivery of TB drugs [3,12]. Lipid-based micro- and nanocarriers have gained popularity owing to the promising potential for their use in TB therapies to enhance the therapeutic efficacy of these highly lipophilic drugs by improving their solubility and consequently enhancing their bioavailability, while drug release can also be better controlled [7,12,13,14,15]. The use of micro- or nanotechnology allows for the careful engineering of dosage forms to target specific sites or cells within the body, such as phagocytic cells that are infected with mycobacteria [3,16]. Several methods exist for targeted drug delivery, including the encapsulation of drugs or chemical conjugation. Dendrimers, liposomes, polymeric nanoparticles, micelles, nanogels, and solid lipid particles are various forms of carriers that offer the potential to encapsulate both lipophilic and hydrophilic TB drugs to enable various routes of administration [3,7,14]. Oil-in-water (o/w) or water-in-oil (w/o) dispersions, for example, micro- and/or nanoemulsions that display significant thermodynamic stability, offer an attractive formulation method, since it has been well established that such formulations do improve drug delivery [3].

The formulation of drug delivery systems that are capable of directly targeting the specific site of infection should improve the efficacy and simultaneously decrease toxic effects, as less of the needed active ingredient is required to exert the same therapeutic effect [3,17]. Extrapulmonary TB (EPTB) often occurs without the presence of pulmonary TB (PTB) and accounts for almost 20% of newly diagnosed TB cases. The co-occurrence of EPTB with PTB, on the other hand, accounts for around 6% of newly diagnosed TB cases. However, in most cases, systemic TB involvement often results in EPTB manifestations, including CTB [18]. Currently, CTB treatment follows the same systemic regimen as PTB, with additional surgical interventions that may be required. However, no topical formulations are currently available to adequately treat CTB [19,20,21]. This emphasizes the need for novel drug delivery systems that may be used directly for the treatment of CTB by targeting bacilli found within the skin lesion. Whilst the need for new TB drugs is particularly urgent, attention should also be given to the development of novel drug delivery systems and/or the improvement of existing drug delivery systems to ultimately improve the bioavailability of TB drugs to enhance treatment outcomes [3].

Self-emulsifying drug delivery systems (SEDDSs) have been widely used in drug formulation to improve drug solubility and subsequent drug bioavailability. Despite the evident advantages of SEDDSs, research on their application in topical and transdermal drug delivery is still limited [22,23]. SEDDSs are associated with relatively inexpensive manufacturing methods and have the advantage of enhancing the stability of typical emulsions comprising highly lipophilic drugs. Additionally, SEDDSs may protect drugs that are susceptible to hydrolysis [24,25]. SEDDSs are generated by combining an oil phase containing the dissolved lipophilic drug and a surfactant phase, which usually includes a combination of a surfactant and a cosurfactant. Upon the addition of the water phase, spontaneous o/w or w/o emulsions may form without the addition of kinetic energy (increased heat (>37 °C) and/or excessive stirring), and these may then be further characterized as micro- or nanoemulsions [23,26,27]. For dermal SEDDSs, one requirement for the formation of these SEDDSs is that minimal water is necessary to enable spontaneous emulsion formation as a result of the limited aqueous volume available in the skin. What is more, when seeking to include a hydrophilic drug, the said drug must first be dissolved in the water phase, which is then gradually introduced into the oil–surfactant concentrate to form a final emulsion (be it an emulsion, microemulsion, or nanoemulsion) before application. In this case, emulsions are formed, and not SEDDSs. The self-emulsification process is only utilized to form these dermal formulations that contain a hydrophilic drug. Regrettably, no standardized characterization has yet been developed for dermal formulations prepared in this manner [22,23,24,25,26,27].

The inclusion of safe and effective penetration enhancers that can alter the structure of the lipophilic stratum corneum (SC) of the skin or act as a carrier vehicle for drug particles into or through the SC layer has become a popular research topic, specifically in the formulation of topical and/or transdermal drug delivery systems [28,29,30]. Natural and essential oils are well-known penetration-enhancers, owing to their ability to temporarily alter the SC, which in turn allows increased drug permeation [28,29,30]. These oils are generally derived from various parts of trees or plants and, among others, often contain high concentrations of fatty acids. Depending on the degree of saturation, fatty acids are classified as monounsaturated fatty acids, polyunsaturated fatty acids, and saturated fatty acids. These components improve drug solubility within the SC by means of lipid fluidization within the membrane [28,30,31]. Furthermore, natural and essential oils can improve the penetration of both lipophilic and hydrophilic drugs in and through the SC with additional therapeutic benefits, such as anti-inflammatory, antiseptic, and antibacterial properties [32,33,34]. These oils typically contain various components that mostly comprise esters, aldehydes, ketones, phenols, terpenes, and terpenoids, which have all been found to enhance drug penetration through the skin [28,31,35,36]. For example, terpenoids are further divided according to the number of carbon atoms in the structure [37,38]. Monoterpenes (C_10_) comprise two isoprene units and are found in approximately 90% of essential oils [32,35]. The most common monoterpenes in essential oils include limonene, nerol, α- and β-terpineol, α- and β-pinene, and 1,8-cineole [37,38,39]. Limonene, for example, is found in the stems and rinds of citrus fruits and significantly enhances drug the permeation of both hydrophilic and lipophilic drugs [31]. Due to these characteristics, as well as the fact that there is an increasing consumer demand for more natural products, as they are regarded as safer and less toxic compared to synthetic compounds, the inclusion of natural and essential oils in dermatological formulations has gained favor [32]. However, essential oils pose difficulties during the formulation of products due to their generally volatile nature, poor solubility, chemical and thermal instabilities, and possible allergic reactions. The exposure of essential oils, especially monoterpenes, to the air may induce oxidative deterioration that is enhanced with exposure to light or heat [40,41]. Nevertheless, the encapsulation of these oils using nanocarriers, such as emulsions, liposomes, or soft nanoparticles, can help counteract this degradation [42,43]. For these reasons, careful consideration was given to the selection of the oils included in this study. The selected oils (olive oil, frankincense oil, lemon oil, rose blend fragrance, and tea tree oil) were chosen based on their known safety profiles for use on the skin (no significant sensitivity is shown in the concentrations included), their solubilization capacity, and their penetration enhancement abilities.

Furthermore, a surfactant and cosurfactant combination is also generally included in SEDDSs, emulsions, microemulsions, and nanoemulsions to decrease the surface tension between the oil and aqueous phases and, subsequently, enhance the overall stability [44,45] of these formulations and improve the solubility of the included drugs [46,47]. Moreover, nonionic surfactants, including Span^®^83 and Tween^®^60, are commonly used in pharmaceutical formulations, especially dermal products, due to their ability to improve drug solubility profiles through weak hydrophilic and hydrophobic groups present in their structures [46,47]. There are many benefits associated with using non-ionic surfactants, including good compatibility profiles, low cost, and lower toxicity risks compared to ionic surfactants [48]. Also, hydrophile–lipophile balance (HLB) values are allocated to all surfactants and are used as a numerical indicator of the hydrophilic–lipophilic correlation between the groups of the specified surfactant. The water solubility of a surfactant is directly linked to the HLB value, where a high HLB value specifies high water solubility [46,49]. Subsequently, the application of surfactants is governed by the individual HLB values; for example, surfactants with an HLB value between 8 and 18 preferably form oil-in-water (o/w) emulsions, while HLB values between 3 and 6 are more suited for the formation of water-in-oil emulsions (w/o). HLB values ranging from 7 to 9 are indicative of wetting agents that possess both hydrophilic and lipophilic properties [46,50]. Typically, nonionic surfactants possess higher HLB values with optimal properties to form spontaneous o/w emulsions and undergo rapid dispersion when they encounter the aqueous phase [26,50]. Additionally, a combination of two or more surfactants with contrasting HLB values is not uncommon and is frequently adopted to increase the selection range of excipient combinations when formulating to obtain more stable formulations [46,51]. Sorbitan sesquioleate (Span^®^83), with a reported HLB value of 3.7 [52,53], and polyoxyethylene sorbitan monostearate (Tween^®^60), with an HLB value of 14.9 [54], have been selected for this study to improve the stability of the formulated emulsions. Furthermore, the use of a cosurfactant assists in decreasing the surface tension, while ensuring that the overall surfactant phase concentration remains low so as not to irritate the skin [55].

Therefore, this study focused on exploring the possibility of formulating topical emulsions using the self-emulsification mechanism. These formulations comprised an INH and RIF FDC for the treatment of CTB, and incorporated the oils chosen (olive oil, frankincense oil, lemon oil, rose blend fragrance, and tea tree oil), as well as a surfactant and cosurfactant combination consisting of Span^®^83 and Tween^®^60, which are used specifically in dermal formulations to decrease the surface tension between the oil and water phases once the emulsions form through the self-emulsification process. These formulations were subsequently characterized according to standard experiments normally used to describe oral SEDDSs, since no tests have yet been standardized for dermal formulations prepared in this manner. Thereafter, they were inspected to determine the most suitable formulations for topical/transdermal drug diffusion based on their individual characteristics.

## 2. Materials and Methods

### 2.1. Materials

INH and RIF were purchased from DB Fine Chemicals (Pty) Ltd./DB (Sandton, South Africa). Tween^®^60 (polyoxyethylene sorbitan monostearate or polysorbate 60) and Span^®^83 (sorbitan sesquioleate) were procured from Sigma-Aldrich Chemistry GMbH (Steinheim, Germany). The different oil phases tested, that is, olive oil (OLV), frankincense oil (FRK), lemon oil (LEM), rose blend fragrance (RBF), and tea tree oil (TTO), were acquired from Scatters Oils (Johannesburg, South Africa). Distilled water was obtained through a Rephile Bioscience Ltd. system (Boston, MA, USA).

### 2.2. Preformulation Experiments

#### 2.2.1. Preliminary Preparation of Placebo Dermal Formulations Using the Self-Emulsification Mechanism

To determine the feasibility of the various essential oils included in this study to form acceptable dermal formulations, placebo formulations were first prepared and visually examined for any signs of physical instabilities. The surfactant phase was prepared by mixing the surfactant and cosurfactant (Span^®^83 and Tween^®^60) in a 1:1 ratio. It has previously been determined that self-emulsification is not only conditional on the selected oil–surfactant combination, but it also depends on the surfactant phase concentration and the ratio in which the surfactant–cosurfactant is included. Results have also demonstrated that only meticulously chosen excipient mixtures can lead to the spontaneous formation of emulsions [56,57,58,59]. Consequently, the surfactant phase was fixed at a 1:1 concentration ratio, as the literature has shown that more stable emulsions are formed when this ratio is used. In addition, higher ratios have been concluded to expand the emulsion range but cause lower stability that could generate the precipitation of an included drug [22,26,59,60,61,62]. After the preparation of the surfactant phase, a selected oil phase was added, and the mixture was gently stirred, forming SEDDS concentrates.

Nine SEDDS concentrates were prepared for each oil–surfactant combination, comprising varying surfactant-to-oil ratios of 9:1, 8:2, 7:3, 6:4, 5:5, 4:6, 3:7, 2:8, and 1:9. Subsequently, water was slowly incorporated into each SEDDS concentrate using the water titration method until a constant state of milkiness or turbidity was noted. This method is also used to construct pseudoternary phase diagrams [22,57,58,59,60,61,62,63,64,65,66,67]. These formulations were then stored at room temperature (25 °C ± 0.5 °C) and visually inspected after 24 h for any physical instabilities.

#### 2.2.2. Solubility Determination

A rotating solubility bath with a fixed rotating axis was used to determine the solubility of INH and RIF separately in the selected oil phases and purified water. The water bath was set to a temperature of 37 °C ± 0.5 °C. Each sample was prepared in triplicate, where an excess amount of either INH or RIF was weighed directly into 15 mL plastic centrifuge tubes. Next, 100 mg of glass beads were added to each centrifuge tube, facilitating proper dispersion of the solvent and drug particles while rotating in the water bath. Following, 10 mL of the relevant solvent was added to the tubes, and the final samples were tightly sealed with Parafilm^®^. Each centrifuge tube was placed in a water bath that was set to rotate for 24 h. Subsequently, the samples were removed, and the supernatant was withdrawn and filtered through a 0.45 µm Millipore^®^ filter. These samples were adequately diluted with HPLC-grade methanol preceding analysis [23,62].

The final concentrations of INH and RIF dissolved within the different solvents were quantified using the published and validated HPLC method for RIF/INH capsules, as described in the USP [68]. A Hitachi Chromaster HPLC system (Hitachi High-Tech Science Corporation, Tokyo, Japan) equipped with a solvent delivery module, a diode array detector (DAD), a column oven, a temperature-controlled autosampler, and a quaternary pump system was used.

This method utilized a gradient system with an aqueous buffer solution (composed of 1.4 g dibasic sodium phosphate in 1 L of water, adjusted with phosphoric acid to a pH of 6.8) and acetonitrile. Mobile phase A consisted of the buffer solution and acetonitrile in a 96:4 (*v*/*v*) ratio, while mobile phase B comprised acetonitrile and the buffer solution in a 55:45 (*v*/*v*) ratio. A flow rate of 1.5 mL per minute was set. Table 1 provides a summary of the gradient program used during the analysis.

A 150 × 4.6 mm^2^, 5 µm Luna^®^ C18-2 column (Phenomenex^®^, Torrance, CA, USA) was employed during the analysis at 25 °C. The injection volume for the test and reference sample solutions was 20 µL, and the detection wavelength was set at 238 nm. The suitability of this validated method for its intended use has been demonstrated by performing analytical method verification in accordance with the International Council for Harmonization (ICH) analytical validation guideline Q2 [69]. Table 2 summarizes the validation parameters and results obtained.

#### 2.2.3. Simultaneous Thermal Analysis (STA)

STA thermograms of INH and RIF raw materials were assessed with a Mettler DTG 3+ (Mettler Toledo, Greifensee, Switzerland) analyzer. Powder samples of INH and RIF that weighed approximately 5–8 mg were placed in separate open 100 μL aluminum cells. These aluminum cells were heated at a rate of 10 °C/min to a final temperature contingent on the melting point of RIF—thus, not greater than 200 °C, since this is the maximum melting point of the said drug. A nitrogen gas flow of 35 mL/min was retained.

#### 2.2.4. Isothermal Microcalorimetry

The chosen drugs and excipient interactions (compatibility) were established employing a 2277 Thermal Activity Monitor (TAM III) (TA Instruments, New Castle, DE, USA), fitted with an oil bath with an observed stability of ±100 µK over a period of 24 h, and a constant temperature of 40 °C was maintained. A baseline or theoretical response was first determined by measuring the heat flow of 100 mg samples of each component, whereafter the theoretical responses to the measured calorimetric output of the different combinations (i.e., drugs and excipients) were compared to establish compatibility. Interactions or incompatibilities may be indicated if significant variations are detected between the baseline and the measured output. For this study, an integration integral inception of 4 J/g was set. Therefore, any value that exceeds this integral suggests possible interactions between the components [70,71,72].

#### 2.2.5. Construction of Pseudoternary Phase Diagrams

The water titration method was used [22,57,58,59,60,61,62,63,64,65,66,67] to compose pseudoternary phase diagrams for the selected excipients (different oil phases and surfactants). These diagrams were then utilized to identify the optimal concentration combinations for these excipients that would projectively allow stable dermal formulations [60,63]. First, as stated previously, the “surfactant phase” was prepared by combining the surfactant (Span^®^83) and the cosurfactant (Tween^®^60) in a 1:1 ratio, as the literature found that this ratio can be considered most suitable for forming stable emulsions. It has been found that higher surfactant ratios often result in larger self-emulsification areas; however, the resulting formulations are less stable and could facilitate drug precipitation in these formulations [22,26,59,60,61,62]. The surfactant phase was moderately heated (35 °C ± 0.5 °C) and incessantly stirred on a magnetic stirring plate for approximately 30 min to warrant the formation of a homogeneous surfactant mixture. For each oil and surfactant phase combination, SEDDS concentrates across a range of surfactant phase-to-oil ratios (9:1, 8:2, 7:3, 6:4, 5:5, 4:6, 3:7, 2:8, 1:9) as well as one surfactant phase-to-water ratio (1:9) were prepared through gentle stirring and slight heating (35 °C ± 0.5 °C) using a magnetic stirring plate for 30 min, whereafter the water phase (or oil phase specifically for the surfactant phase-to-water) was added in small increments through titration at ambient temperature (25 °C ± 0.5 °C). At the first sign of turbidity or milkiness, no additional water (or oil) was added, and this point was considered the endpoint. For this study, a ratio of surfactant phase-to-water of 1:9 was chosen to determine the minimum amount of oil needed to produce an emulsion with a minimum amount of surfactant phase. The endpoints of the different formulations were plotted using Triplot v1-4 software (Informer Technologies, Inc., Los Angeles, CA, USA) to obtain the final pseudoternary diagrams that identified the specific area of spontaneous emulsification for the specific oil tested [23,50].

### 2.3. Topical Formulation Preparation

Topical formulations were prepared in agreement with the identified checkpoint formulations after the pseudoternary phase diagrams were constructed. The surfactant phase was prepared first by mixing equal parts of Span^®^83 and Tween^®^60 to achieve a 1:1 ratio. This mixture was slightly heated (35 °C ± 0.5 °C) and blended for approximately 30 min with a magnetic stirrer to ensure homogeneity. Following, the individual oil phases were produced by accurately weighing the required amount of RIF and dissolving it in the measured amount of oil to obtain a supersaturated theoretical final concentration of 0.6% (*w*/*v*), as discussed in Section 3.3. Likewise, the aqueous phase that included the INH was composed to comprise a final concentration of 0.2% (*w*/*v*) INH. All oil and water phases were separately subjected to sonication for 5 min to allow the individual drugs to fully dissolve in the respective solvents. The individual oil and surfactant phases were combined and agitated for an additional 30 min, warranting the formation of homogeneous SEDDS concentrates. Lastly, a predetermined amount of water was slowly added in small increments. The dermal formulations obtained (Table 3) were stored at room temperature (25 °C ± 0.5 °C) for 24 h, after which each formulation was visually inspected for phase separation to detect any instability of the formulations [22,23,50,67]. After the characterization experiments were conducted, selected formulations, which were considered physically stable, were freshly prepared before the encapsulation efficiency, assay, drug release, and drug diffusion were determined.

### 2.4. Formulation Characterization Experiments

#### 2.4.1. Zeta Potential, Droplet Size, and Size Distribution

The zeta potential of each placebo topical formulation was measured at 25 °C utilizing a Zetasizer Nano^®^ZS (Malvern^®^ Instruments Ltd., Worcestershire, UK) through dynamic light scattering techniques. Droplet size and size distribution were visually evaluated with a ZEISS LSM 980 confocal laser scanning microscope with an Airyscan 2 detector (Carl Zeiss, Oberkochen, Germany) using a 63× oil objective and the pinhole set at 48 µm, excited with the 405 nm laser. ZEN 3.4 (blue edition) microscopy software was used to enable observation of the individual droplet sizes of each formulation.

#### 2.4.2. Investigating Robustness to Dilution

Dilutions (100-fold) of all formulations were prepared with various solvents comprising different pH values. Purified water and phosphate buffer solutions (PBS—pH 5 and pH 7.4) were used as the different vehicles. A pH value of 5 was implemented to simulate the pH of the outer surface of the skin, while a pH of 7.4 represented the physiological pH. The resulting dilutions were stored at ambient temperature (25 °C) for 24 h prior to visual inspection for phase separation [22,73,74].

#### 2.4.3. Establishing Self-Emulsification Efficacy and Time

Efficacy and self-emulsification time were determined with a type II Distek 2500 dissolution system (Distek, North Brunswick, NJ, USA). A 1 mL sample of each formulation was added to 100 mL of distilled water. The paddles were set to rotate at a speed of 50 rpm for the mild agitation of these diluted formulations, while the water was maintained at 32 °C (±0.5 °C), which is the average temperature of the skin surface. The diluted emulsions were visually monitored, and the time was taken until a homogenous dispersion formed. This time was consequently noted and used to establish the efficacy of spontaneous emulsification by grading each formulation according to Table 4 [22,23,27,50,62,74].

#### 2.4.4. Viscosity Measurement

A Brookfield^®^ Viscometer model DV2T LV (Brookfield Engineering Laboratories, Inc., Stoughton, MA, USA), equipped with a Brookfield^®^ temperature controller attached to a circulating water bath (25 °C ± 0.5 °C), was used to take viscosity measurements. Different spindles (T-Bar D LV, T-Bar E LV, and T-Bar F LV) were employed, which were set at 5 rpm to ensure optimal torque values. A total of 30 viscosity measurements (every 10 s for 5 min) were recorded, and a subsequent average for each formulation was calculated [22,62].

#### 2.4.5. Cloud Point Establishment

Each formulation was diluted (1:100) with distilled water in a glass beaker and placed in a water bath. The initial temperature was set at 25 °C and increased in increments of 2 °C every 60 s until a cloudy or murky appearance of the diluted dispersion was observed [22,27]. This change in appearance (the cloud point) is defined as the point where dehydration of the included excipients transpires [22,62,75].

#### 2.4.6. Thermodynamic Stability Experiments

Each topical formulation tested was subjected to six heating (45 °C) and cooling (4 °C) sequences for at least 24 h. These formulations were continuously visually examined for any conceivable phase separation or drug precipitation that may have occurred [23,50]. Additionally, each formulation was centrifuged for 30 min at 3000 rpm, whereafter they were again optically examined for any signs of physical instability, including cracking, drug precipitation, creaming, and/or phase separation [23,67,76].

#### 2.4.7. pH Determination

A Mettler^®^ Toledo pH meter equipped with a Mettler^®^ Toledo InLab^®^410 NTC electrode 9823 (Mettler^®^ Toledo International Inc., Columbus, OH, USA) was employed to determine the pH values of the different formulations evaluated. The probe was calibrated at a pH of 4 and 10 before any measurement occurred.

#### 2.4.8. Encapsulation Efficiency (%EE)

Ultracentrifugation was applied using an Eppendorf^®^ 5804 R centrifuge equipped with an A-4-44 rotor (Merck, Modderfontein, South Africa) to determine the %EE of each formulation. In this study, %EE was defined for each drug as the phase in which it was supposed to be dissolved. As stipulated in the methodology, all formulations that were deemed acceptable were centrifuged at 25,000 rpm (25 °C) for 45 min. Care was taken not to centrifuge at the highest speed of the apparatus so as not to “break” the emulsion droplets and subsequently release the encapsulated drug in the dispersed phase. A 25 g sample was placed in an Eppendorf tube^®^ and subjected to centrifugation at 25,000 rpm (25 °C) for a total of 45 min. Thereafter, the resulting supernatant was extracted, diluted, and transferred to HPLC vials (in triplicate) to determine the unentrapped amount of INH and RIF. The unentrapped INH and RIF were subsequently subtracted from the initial added amount, and the %EE was calculated [22,50].

#### 2.4.9. Assay

The prepared formulations were analyzed to determine the drug content (%). First, a phosphate buffer solution (PBS) was composed by dissolving 7 g of Na_2_HPO_4_ in 5 L of ultrapure water (Milli-Q). The pH was adjusted to 6.8 using phosphoric acid. Following this, the buffer solution was mixed with methanol in a 94:6 ratio to obtain a solvent solution. Approximately 5 g of each formulation was diluted to 50 mL with the solvent solution and then filtered through a 0.45 μm membrane filter into HPLC vials. The samples were analyzed in triplicate, whereafter the % drug content was calculated.

### 2.5. Topical Delivery

#### 2.5.1. Drug Release Studies

The ability of the formulations to release INH and RIF was determined through drug release studies, which were performed according to the method followed for skin diffusion experiments (next section). For these experiments, polytetrafluoroethylene membranes were utilized in the place of human skin, which were carefully placed between the donor and receptor compartments of vertical Franz cells. Drug release studies were carried out over a period of 6 h, with hourly extractions from receptor compartments (n = 10) [22,77].

#### 2.5.2. Preparation of Skin Samples

The full-thickness skin used for diffusion studies was donated by anonymous Caucasian female donors who underwent abdominoplastic surgery at different hospitals in South Africa. These skin samples are considered biological waste materials. The researchers did not have contact with these skin donors or the physicians who performed the surgeries at any time, and an independent person (who also deidentified all patient details) procured these samples from the various hospitals after informed consent was granted by the said donors. Prior to the skin diffusion studies, ethical approval was granted by the Ethics Committee of the North-West University (Ethics number: NWU-00111-17-A1-07) for the collection, transportation, and handling of skin samples. Upon collection, the full-thickness skin was frozen and stored at –20 °C for a maximum of 6 months in the biosafety laboratory until use.

Before investigation, the frozen skin was removed and allowed to thaw at room temperature (±25 °C). The skin was visually examined for any defects that could affect the skin integrity and ultimately influence drug permeation, for example, large hair follicles, stretch marks, or any visible injury to the skin. The skin was then dermatomed using a Zimmer^TM^ electric dermatome, model 8821 (Zimmer^TM^ Ltd., Swindon, Wiltshire, UK) to separate the dermis, epidermis, and SC from the other layers. Dermatomed skin samples, with a thickness of 400 µm, were placed on Whatman^®^ filter paper, with care being taken to smooth the skin and to ensure that it was flat on the filter paper. The filter paper was consequently wrapped in aluminium foil and frozen (–20 °C) until needed within 24 h [22,78,79].

#### 2.5.3. Skin Diffusion Studies

Before each in vitro skin diffusion experiment, the dermatomed skin was thawed and carefully cut into circles (15 mm) to fit between the receptor and donor compartments of the vertical Franz diffusion cells (n = 10). The receptor compartment contained a 2 mL mixture of PBS at a pH of 7.4 and methanol in a 9:1 ratio, which was continuously stirred with a magnetic stirrer at 720 rpm. A thin layer of Dow Corning^®^ high-vacuum grease was spread around the surface of each compartment before the cut skin circles were carefully placed on top of each receptor compartment, with the SC facing upwards. Each donor compartment was cautiously placed on top of the skin circle, and a thick layer of vacuum grease was spread around the edges of the vertical Franz diffusion cell to ensure a tight seal and to prevent leakage from the cell. Thereafter, the donor compartments were fastened with a horseshoe clamp to ensure the two compartments were held together.

A 1 mL sample of a selected topical formulation was consequently placed in the donor compartment, which was then covered with Parafilm^®^ and secured with a plastic cap to prevent evaporation of the said formulations. The vertical Franz diffusion cells were cautiously placed on a tray and transferred to a water bath (37 °C ± 0.5 °C to simulate the systemic circulation) containing a Variomag^®^ stirring plate, set at 750 rpm. Each receptor phase was fully extracted at 2 h intervals and replaced with a new PBS/methanol solution at an equilibrated temperature for a complete 12 h cycle. The extracted receptor compartment samples were filtered with a 0.45 µm filter and analyzed in triplicate utilizing a validated HPLC method [22,77,78,79].

#### 2.5.4. Tape Stripping

After the 12 h skin diffusion experiments were completed, the skin circles were carefully removed from the individual Franz diffusion cells and mounted on a board covered with Whatman^®^ filter paper. The residual formulation was gently wiped off the surface of each skin circle. Next, the SC–epidermis layers were removed using clear Scotch^®^ magic tape strips. One tape strip was used to clean the skin and was discarded before 15 additional strips were used to remove the indicated skin layers. Once the entirety of the SC–epidermis is removed, the skin should have a glistening appearance. For each Franz diffusion cell, the 15 tape strips were placed in a polytop vial while the remaining skin sample, specifically the diffusion area, was cut into smaller pieces and placed in a separate polytop vial. A volume of 5 mL solvent was subsequently added to each polytop, and all samples were refrigerated for a minimum of 24 h at 4 °C before analysis. The resulting solvent was filtered and analyzed in triplicate using a validated HPLC method [22,77,78].

### 2.6. Determination of INH and RIF Stability

The degradation of INH and RIF over 24 h and their stability across a range of temperatures were measured by preparing a standard mixture comprising 20 mg of INH and 40 mg of RIF. This mixture was made up to a 250 mL volume in a volumetric flask with a solvent comprising a Na_2_HPO_4_ buffer solution (pH 8) and HPLC grade methanol in a 96:4 ratio. From this solution, three samples were filtered through a 0.45 µm Millipore^®^ filter and stored for 24 h at different temperatures, namely, 4 °C, 25 °C, and 40 °C. Another sample was also filtered and immediately injected (t = 0) into HPLC vials for subsequent analysis. Following the 24 h period, the three samples stored at different temperatures were filtered and analyzed by means of HPLC, whereafter the resulting drug concentration in each sample was calculated.

## 3. Results and Discussion

### 3.1. Preformulation Studies

#### 3.1.1. Placebo Dermal Formulations Prepared Using the Self-Emulsification Mechanism

The oils chosen for the preparation of the dermal formulations were olive oil (OLV), frankincense oil (FRK), lemon oil (LEM), rose blend fragrance (RBF), and tea tree oil (TTO). The formulations that comprised OLV, FRK, and LEM displayed the highest physical stability profiles, whereas formulations consisting of RBF and TTO were the least stable due to phase separation. All RBF formulations with a surfactant concentration of 40% or lower showed clear phase separation. Formulations with TTO also demonstrated physical instability through phase separation, except for one formulation with a 1:1 ratio of surfactant-to-oil phase. Consequently, it was decided not to continue with formulations that consisted of TTO. Similarly, various rose RBF formulations demonstrated instability at low surfactant concentrations (<50%). Although formulations that included higher surfactant phase concentrations (>50%) showed improved stability, it is noted that increased surfactant concentrations are often associated with skin irritation [22,80,81,82,83]. For this reason, all formulations containing RBF as the oil phase were also excluded from further studies.

Interestingly, most of the OLV, FRK, and LEM formulations were stable, with phase separation only observed in formulations with surfactant concentrations less than 20%. These instabilities indicate that the interfacial layer energies are not lowered effectively between the immiscible phases due to the low surfactant concentrations [84,85].

#### 3.1.2. Solubility

The inclusion of natural oils to improve drug permeation through the skin has been utilized for many years to enhance the delivery of lipophilic and hydrophilic drug molecules through the various skin layers. These chemical penetration enhancers temporarily alter the complex skin structure to allow drug permeation, or act as drug carriers across the various skin layers [28,29,30,31,86].

INH is a white crystalline powder [87] that is soluble in water to a degree of 125 mg/mL at 25 °C ± 0.5 °C, but its permeability has been described as limited [88,89,90]. The aqueous solubility of INH at a pH of 6.56 and a temperature of 37 °C ± 0.5 °C has been recorded as 152.02 mg/mL [91] or 174, 161, and 153 mg/mL at a pH of 1.2, 4.5, and 6.8, respectively [92]. In this study, the solubility of INH in water was 100.11 mg/mL at pH 7, while its solubility in the selected oils was found to be very low, as expected, where INH concentrations could not be quantified in any of the selected oils using HPLC analysis. The solubility of INH fluctuates according to the pH of the solvent, and its solubility has been found to increase as the pH of the solvent decreases, possibly due to the protonation of INH that occurs in more acidic solvents [5,74].

RIF, on the other hand, possesses relatively low aqueous solubility (0.10 ± 0.01 mg/mL), and its solubility varies depending on the pH of the selected solvent, where RIF has shown higher solubility in acidic solvents (for example, gastric pH of 1–3), but has also been found to be more unstable at pH levels lower than 6 [87,88,93]. Its solubility can, however, deviate to a factor of up to 100. Alves et al. [94] reported that the solubility of RIF decreased significantly from 100 mg/mL to 4 mg/mL when the pH of the aqueous environment was adjusted from a pH of 2 to a pH of 5.3. Moreover, the solubility was reduced to 2.8 mg/mL in a solvent with a pH of 7.5. RIF absorption occurs primarily in the duodenum (pH 4–6), but in this area, its solubility is notably lower [94].

The solubility of RIF in OLV was markedly higher (0.46 ± 0.12 mg/mL) compared to its solubility in water. However, it was not as high as expected. OLV mainly comprises fatty acid components, including oleic acid (approximately 76–77%), palmitic acid (11–12%), and smaller amounts of linoleic, stearic, linolenic, and palmitoleic acids [31,78,95,96,97]. This fatty acid content of OLV is responsible for the lower pH value reported at about 5.17 at ambient temperature. Furthermore, it has been observed that when OLV samples are stored at 25 °C for 30 days, the pH of the oil decreased to approximately 4.49 as the free oil acidity (% oleic acid) increased from 0.28 to 0.63% [97]. Thus, RIF depicted limited solubility in OLV as a result of the lower pH of this oil, and the fact that possible degradation occurred during the 24 h period.

The use of essential oils appeared to significantly improve the solubility of RIF, since the RIF concentrations in LEM and FRK were found to be 2.14 ± 0.12 and 2.32 ± 1.09 mg/mL, respectively. However, these results were again not as favorable as expected. It is well-recognized that essential oils are significantly sensitive to light, oxygen, humidity, and high temperatures, especially constituents such as terpenes and terpenoids, which are mainly part of these oils. Subjection to some of these environmental factors during solubility testing could not entirely be prevented. Moreover, the oxidation of essential oils increases with exposure to ultraviolet and visible light, resulting in the formation of alkyl radicals [98,99,100]. In a study performed with LEM, it was found that when LEM is exposed to visible light, a noticeable decrease in the monoterpene concentration was observed, as these structures degrade rapidly under light exposure. Furthermore, chemical reactions were disclosed to occur twice as fast when a temperature increase of only 10 °C was initiated [99]. Temperature is a known chemical degradation facilitator, particularly in auto-oxidation and decomposition, where free radicals are formed in essential oils [100]. During solubility testing, all samples were exposed to visible light and a temperature of at least 37 °C that could have promoted the degradation and oxidation of these essential oils, as no chemical interactions were seen in the OLV samples. From the results obtained, it is clear that FRK and LEM can meaningfully increase the solubility of RIF compared to OLV; however, the solubility in these oils could also have been restricted as a result of the possible decomposition of these oils during testing, since not all environmental factors could be effectively controlled.

#### 3.1.3. Isothermal Drug-Excipient Compatibility and Stability Studies

Drug-excipient compatibility studies are performed to classify, quantify, and predict potential interactions in order to establish whether a selected combination of a drug and an excipient can form a stable formulation [101]. Furthermore, the effects of water and temperature on drug–excipient combinations during formulation can also be evaluated. During these isothermal drug–excipient compatibility studies, the drug(s) and excipients are simply blended to form physical mixtures in predetermined ratios that are then exposed to certain stress conditions. Various analytical methods can subsequently be used to determine the physicochemical characteristics of these combinations [101,102,103,104,105,106,107].

**Figure 1:**
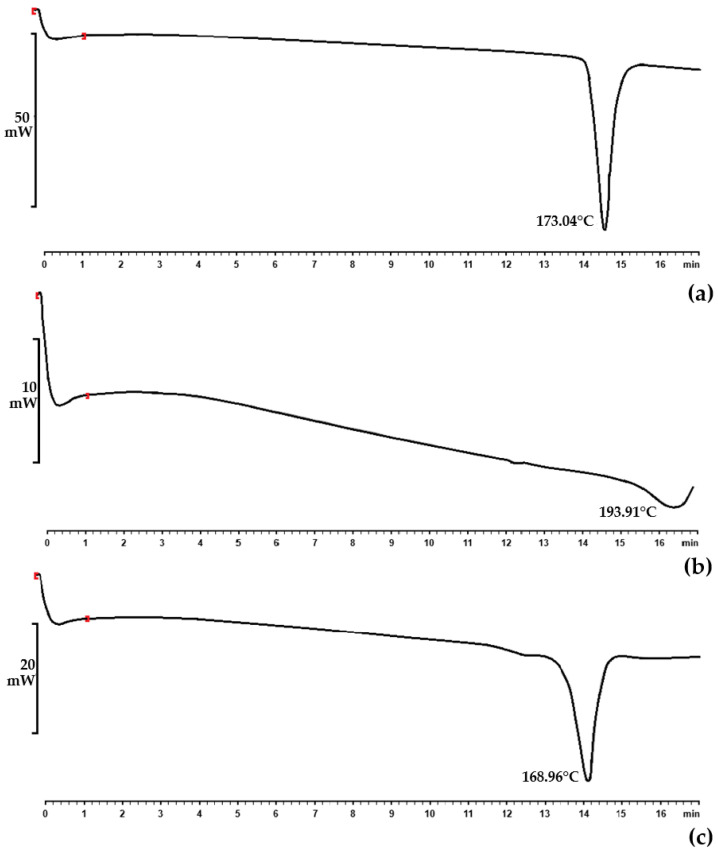
DSC thermograms of (**a**) INH raw material displaying a melting point of 173.04 °C; (**b**) RIF raw material with a melting point of 193.91 °C; and (**c**) the physical mixture of INH and RIF showing a melting point of 168.96 °C.

The compatibility between INH, RIF, the surfactant phase, and the selected oil phases was examined using STA and isothermal microcalorimetry. The thermogravimetric analysis part of the STA revealed that the samples did not undergo substantial weight loss or gain. Subsequently, differential scanning calorimetric thermograms were obtained for INH (Figure 1a), RIF (Figure 1b), and a mixture of the active ingredients (Figure 1c).

Individual endothermic events for INH and RIF were observed at 173.04 °C and 193.91 °C, respectively. The endothermic event of INH in this experiment corresponds to the melting point in the literature for this drug, that is, 170–174 °C [87]. RIF, conversely, exists in two polymorphic forms (Form I and Form II) that show different physicochemical characteristics [105]. The literature has indicated that Form I have a lower chemical stability at higher temperatures compared to Form II. Form I directly decompose, which is normally shown by a sharp exotherm at 255–266 °C [105,106,108]. It exhibits amorphous behavior, while Form II comprises elongated crystals [106]. Moreover, Form II, which is mainly the commercially available form, is considered metastable and melts at 180–197 °C. This is immediately followed by recrystallization to Form I at about 197–223 °C, which is believed to be characteristic of solid–liquid–solid transition. It furthermore finally decomposes at approximately 240–266 °C [105,106,108]. Therefore, the RIF used in this study was polymorphic Form II, which is the more crystalline form.

For isothermal microcalorimetric analysis, an interaction integral inception of 4 J/g was set. An interaction integral signifies an area between the theoretical (zero interaction) line and the calculated calorimetric output. The closer the theoretical measurement is to the calculated output, the smaller the perceived interaction integral, indicating a lack of interaction [70,71,72]. In particular, no interactions between INH and RIF (1.188 J/g) could be established despite reports of significant incompatibility between these two drugs [5,10,103,107,109,110,111,112,113,114]. This lack of interaction is likely attributable to the absence of water and/or an acidic environment necessary to catalyze a reaction between INH and RIF [115].

Prominent changes in heat flow between the drug combinations and selected excipients were observed that exceeded 4 J/g. The largest integration (12.735 J/g) was depicted when OLV was included as the oil phase in the physical mixture containing the selected drugs and the surfactant phase. This was followed by mixtures comprising LEM (11.928 J/g), TTO (11.384 J/g), FRK (11.191 J/g), and RBF (10.926 J/g). However, these values did not differ statistically significantly (*p* > 0.05), indicating that the possible interaction in all combinations is due to the INH and RIF that were dissolved in the liquid phases.

#### 3.1.4. Pseudoternary Phase Diagrams and Topical Formulation Preparation

**Figure 2:**
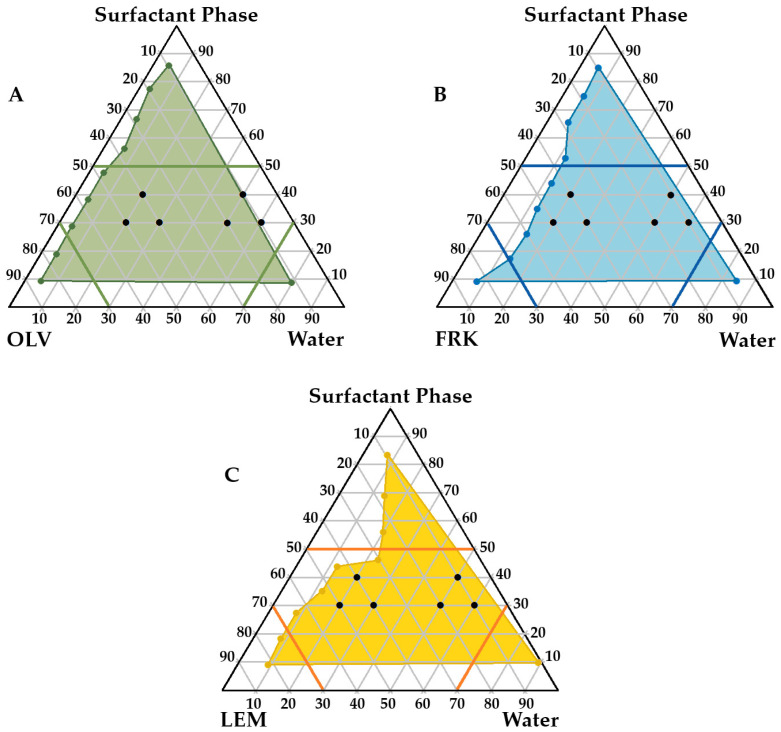
Pseudoternary phase diagrams displaying checkpoint SEDDS formulations for (**A**) olive oil (OLV), INH, RIF, surfactant phase and water; (**B**) frankincense oil (FRK), INH, RIF, surfactant phase and water; and (**C**) lemon oil (LEM), INH, RIF, surfactant phase and water. The colored areas indicate where spontaneous emulsification may occur.

After reflection on the preformulation studies, it was decided to continue with OLV, FRK, and LEM as oil phases, as it was anticipated that the formation of emulsions using these oils could “better protect” RIF from interacting with INH without phase separation occurring. Following, pseudoternary phase diagrams were constructed for the oils mentioned. These diagrams are used to illustrate self-emulsifying regions for specific combinations of excipients [22,116,117], which allow careful selection of optimal excipient ratios to facilitate self-emulsification of the system. Figure 2 shows the notably large areas obtained (illustrated in color) for the OLV, FRK, and LEM SEDDSs where spontaneous emulsification may occur.

These regions were selected to specifically accommodate certain properties considered essential for adequate topical drug delivery. A high concentration of the surfactant phase was avoided, as it can lead to skin irritation. For this reason, it was decided to maintain a surfactant ratio below 5 (as indicated in the diagrams). On the other hand, water-rich zones and oil-rich areas were circumvented because formulation in these regions is likely to result in the formation of micelles or reverse micelles, respectively. These structures are relatively rigid, with decreased deformability, and may negatively affect topical drug delivery. Consequently, it was deemed that the water and oil components of the different emulsions formed should not exceed a ratio of 7 [22]. Six checkpoint formulations within each self-emulsification area of the individual oils were subsequently selected (Figure 2).

All topical formulations chosen comprised the same surfactant:oil:water combination ratios. Formulations derived from the checkpoints were prepared and retained at ambient temperature for 24 h. Visual inspection of the 18 prepared topical formulations indicated that only 10 could be considered stable. Clear signs of instability were displayed, such as phase separation, flocculation, or drug precipitation, in OLV442, OLV352, FRK442, FRK352, FRK343, LEM442, LEM352, and LEM343, and these were therefore considered unsuitable for topical drug delivery. The remaining 10 formulations (Table 5) were subjected to further characterization experiments to determine their feasibility and suitability as topical drug delivery systems for the INH and RIF FDC. These formulations could be described as having a light yellowish color with a cream-like consistency, not varying distinctively.

### 3.2. Characterization of the Formulations Prepared for Topical Drug Delivery

#### 3.2.1. Zeta Potential, Droplet Size, and Size Distribution

Droplet characterization is a crucial component during the development of topical formulations, as the zeta potential, droplet size, and size distribution affect not only the stability of the final formulation, but also drug release and permeability. For the zeta potential, it has been documented that the values should ideally be highly negative (<−30 mV) or positive (>30 mV), because this will cause the droplets to be highly repulsive and, subsequently, avoid coagulation [22,118]. It has also been established that emulsions stabilized by steric and combined electrostatic forces, such as in the case where a surfactant and cosurfactant are included in the formulation (for example, Tween^®^60 and Span^®^83), a minimum zeta potential of –20 mV may also be considered acceptable [22,62]. The negative zeta potential values (Table 5) associated with the selected formulations indicate that all emulsions can be considered stable to highly stable. It also appears that LEM nanoemulsions may be the most stable. The negative potential is due to the presence of free fatty acids contained in the oil phases. However, the net potential of the skin is negative, which theoretically suggests that a decreased affinity between the formulation applied to the skin and the skin itself will probably ensue, leading to reduced drug delivery and penetration [22,119]. Thus, OLV343 could be considered the best formulation in terms of zeta potential.

**Figure 3:**
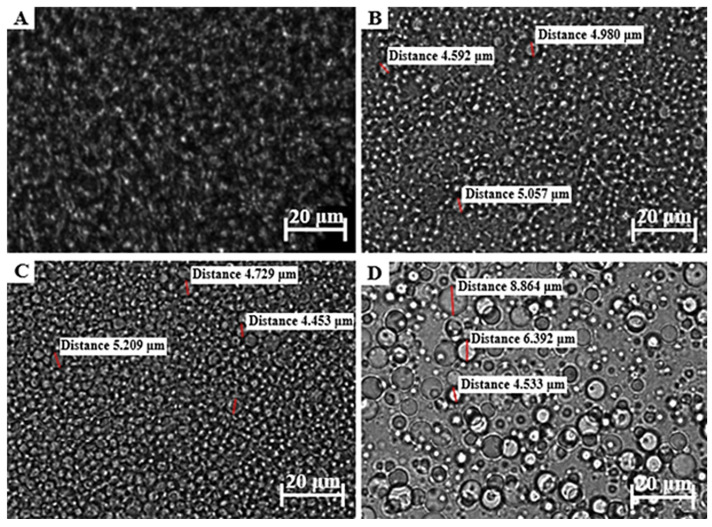
Micrograph of (**A**) OLV343, (**B**) OLV415, (**C**) OLV325, and (**D**) OLV316.

**Figure 4:**
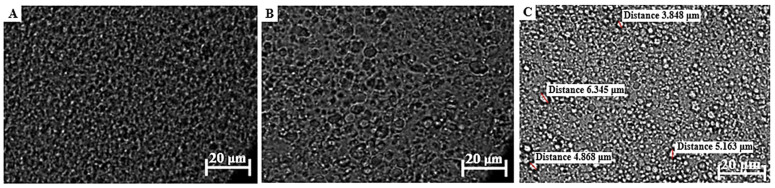
Micrographs of (**A**) FRK415, (**B**) FRK325, and (**C**) FRK316. Individual droplet sizes for FRK415 and FRK325 could not be measured, since no uniform, round droplets could be identified.

**Figure 5:**
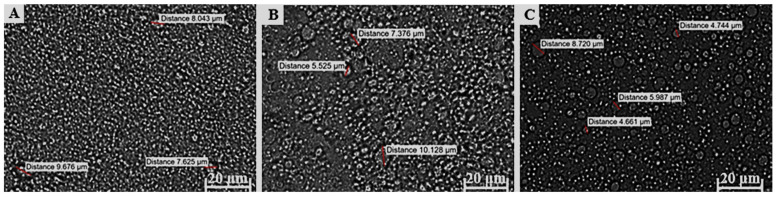
Micrograph of (**A**) LEM415, (**B**) LEM325, and (**C**) LEM316.

The droplet size of an emulsion correlates with the total surface area, where an increase in the droplet size causes a decrease in the surface area, which, in turn, is associated with slow drug release from the formulation and consequently less than ideal drug absorption [23,62,120,121]. Furthermore, an increased droplet size tends to cause emulsion instability by, for example, coalescence. Average droplet sizes larger than 600 nm will normally show poor drug delivery to the deeper skin layers, and will likely accumulate in the SC. To ensure dermal drug penetration, an approximate droplet size of 700 nm or smaller has been suggested [122]. Droplet size and size distribution of the different formulations were visually analyzed with a ZEISS LSM 980 confocal laser scanning microscope. A small sample of approximately 1 μL was carefully placed on a microscope slide before analysis. These micrographs are shown in Figure 3, Figure 4 and Figure 5.

The formulations comprising OLV exhibited a relatively uniform droplet size distribution in OLV343, OLV415, and OLV325 (Figure 3), where the OLV343 droplets showed the best droplet size uniformity. Moreover, OLV343, OLV415, and OLV325, with an average droplet diameter for OLV415 and OLV325 of 4.876 μm and 4.839 μm, respectively, presented the smallest droplet sizes of the 10 formulations. OLV316 presented the largest droplets of all OLV-containing formulations, with an average of 6.596 μm. These differences observed in size measurement are probably due to the droplets being too small to accurately measure size using the confocal microscope, as size is manually determined. Also, due to the placement of microscope slides on the formulations, the droplets were probably “flattened”, thereby “stretching” their dimensions and giving the illusion of possessing larger droplet sizes.

All FRK formulations (Figure 4) also displayed relatively uniform droplet size dispersions. Droplet size could not be measured for FRK415 or FRK325 because these droplets were also considered too small to accurately determine their individual sizes, while FRK316 displayed an average droplet size of 5.056 μm.

An attempt was made to measure the droplet sizes of LEM415; however, as can be seen in Figure 5, the droplet size could also not be measured precisely. The average droplet sizes for the other LEM formulations (Figure 5) were determined as 7.676 μm and 6.028 μm, individually. Interestingly, a decrease in droplet size was observed between LEM325 and LEM316 when the water phase increased above the oil and surfactant phase.

Given the average droplet sizes measured for the selected topical formulations (Table 5 and Figure 3, Figure 4 and Figure 5), all formulations could at the very least be described as being in the micro range. OLV343, FRK415, FRK325, and LEM415 formulations possibly fall within the nano range and, therefore, can probably be described as nanoemulsions. Nonetheless, these findings suggest that OLV343, FRK415, FRK325, and LEM415 will most likely display improved drug release and absorption compared to the other emulsions tested.

The polydispersity index (PDI) for each formulation indicates the droplet size distribution throughout the formulation. Normally, emulsions with low PDI are stable and are often found to correlate with droplet size, where larger droplets yield higher PDI values. The PDI can also be used as a predictor of drug release. Theoretically, smaller and more uniform droplet sizes and distributions will result in improved drug release [77,120,121]. Although the OLV formulations overall depicted the smallest droplet sizes that could be measured, the PDI values for these emulsions indicate that the OLV emulsions are predicted to be less suitable formulations for transdermal drug delivery, as an average PDI value of 0.76 was obtained. These formulations will be more effective for topical use. However, comparatively, OLV343 had the optimal PDI value. Interestingly, it seems that as the water phase increased, an increase in the average droplet size was seen, indicating that the component ratios played a significant role in determining the droplet size. Although no set PDI criteria exist for dermal formulations, generally, a PDI value of 0.7 or higher suggests a large droplet size distribution in the formulation. If the PDI exceeds the overall accepted range of 0.05–0.7, it has been suggested that microscopic techniques should rather be used to properly characterize the average droplet size distribution, since dynamic light scattering can misguidedly identify small, clustered droplets as single large droplets [22,122]. Remarkably, an overall trend was observed in which an increase in droplet size and PDI values resulted in lower zeta potential values, which might indicate comparatively lower formulation stability.

#### 3.2.2. Robustness to Dilution

Upon visual examination of the diluted samples, only OLV343, FRK415, and LEM415 displayed phase separation after 24 h. Important to note, however, is that OLV343 and FRK415 were robust during dilution with purified water, but phase separation occurred once these formulations were analyzed in PBS with varying pH. LEM415 only displayed phase separation in PBS with a pH value of 7.4; however, it was considered stable at a pH of 5. These nanoemulsions were diluted in a 1:100 ratio, which is a significantly large dilution that is highly unlikely to occur when applied to the skin surface, as sweat is the most substantial fluid that will be able to affect the stability of topical formulations. The sweat rate of a healthy person’s body is approximately 500–700 mL daily over the entire surface area [123]. Moreover, no standard has been developed for topical drug delivery systems. Also, it has been suggested that during formulation, the emphasis should rather be on the ability of emulsions to resist phase separation when subjected to varying pH environments, as opposed to the volumes to which the formulations are exposed [22]. Therefore, despite the phase separation observed, the mentioned formulations could not be rejected as unsuitable.

#### 3.2.3. Self-Emulsification Efficacy and Time

For oral SEDDS formulations, the action of spontaneous emulsification has been recognized as the rate-limiting step for absorption, since adequate absorption cannot occur if emulsification does not transpire first [27,62]. On the other hand, topical drug delivery depends on the diffusion rate through the outer layer of the skin, namely, the SC. For this reason, prolonged contact time with the skin is more important, which implies that a short self-emulsification time for topical SEDDSs or formulations prepared through the self-emulsification process is not that imperative, although the process of self-emulsification is still essential [22]. Therefore, in this study, any formulation that presented with a C-grade or lower classification (Table 3) could be judged appropriate. All formulations received a D or E grading, which implies good occlusivity, and were subsequently reasoned to be suitable for further analysis. OLV microemulsions formed notably faster compared to nanoemulsions, which comprised FRK and LEM as oil phases. The FRK and LEM nanoemulsion formulations furthermore displayed similar self-emulsification times, suggesting that enhanced kinetic barriers formed between these excipient combinations as opposed to the OLV microemulsions.

#### 3.2.4. Viscosity of the Formulations

It was previously believed that dermal formulations with high viscosity would result in poor drug delivery as a result of protracted drug release from the formulations. However, it has been established that viscous formulations actually enhance topical drug delivery due to increased occlusive properties [22,77]. For these measurements, it was found that the T-Bar F LV spindle could be used for all formulations, and that none of the viscosity values differed notably with time. The type of oil that was included in the selected formulations in this study had a definite influence on the viscosity of the different formulations. Extended self-emulsification times could furthermore be related to enhanced viscosity, suggesting a potential direct correlation between the ease of spontaneous emulsification and viscosity. Likewise, in general, as the average droplet size increased, a decrease in viscosity was observed. Thus, FRK and LEM nanoemulsions will probably demonstrate improved dermal drug delivery because of their smaller droplet sizes, increased occlusivity, and improved physical stability. According to Stoke’s law, an increase in the viscosity and a decrease in droplet size are directly correlated with a slower droplet movement speed in the system, resulting in a more physically stable emulsion system [124]. Furthermore, higher-viscosity products can be useful in treating localized conditions, since they remain at the site of application. Finally, it was noted that as the oil content decreased and the water phase increased in the emulsions, viscosity decreased, which could compromise the physical stability.

#### 3.2.5. Cloud Points Determined

The cloud point is described as the theoretical temperature that diminishes the ability of SEDDSs to maintain their spontaneous emulsification ability. This is also the point where erratic drug release is found, and where irreversible phase separation may be initiated due to excipient dehydration caused by increased temperatures [22,62,125]. Theoretically, all OLV microemulsions portrayed unacceptable cloud points (<32 °C) and thus excipient dehydration close to body temperature, suggesting possible unpredictable drug release and formulation instability. Conversely, the FRK and LEM formulations did not exhibit cloud points below the skin surface temperature (32 °C), and were thus able to retain their spontaneous self-emulsification properties.

#### 3.2.6. Thermodynamic Stability of the Emulsions

Exposure to various types of kinetic and thermodynamic stressors is an approved method for determining the physical stability of SEDDS formulations [50,125]. Surfactants (and cosurfactants) are normally added to emulsions to lower the interfacial energy between phases to improve the ultimate stability of the indicated formulation. However, this inclusion does not guarantee stable formulation development. Consequently, excipients should be selected to improve solubilization and stabilization, and thus, SEDDSs should theoretically be able to withstand phase separation, creaming, or cracking that would typically render a formulation unstable [46,47,123].

Only OLV415, OLV325, OLV316, and LEM316 were capable of withstanding all the applied thermodynamic and kinetic stress conditions, while the other formulations were considered inapt. Even though FRK415 and LEM325 passed the heating–cooling cycles, the other formulations showed clear signs of phase separation. Most of the SEDDSs that were considered unacceptable also showed signs of cracking and phase separation during centrifugation testing. Nonetheless, based on stability experimentation, OLV325 is considered the most stable formulation.

#### 3.2.7. pH Determination of Topical Formulations Prepared Through the Self-Emulsification Mechanism

The pH range considered acceptable for dermal drug delivery is a range that closely approximates the natural pH of the skin (4.5 to 5.0) [73], which is between 5.0 and 9.0 to avoid skin irritation [22,125,126,127]. All formulations had an average pH value close to 7. The OLV and LEM emulsions, however, showed slightly lower values, but this could not be considered significant. Therefore, all topical formulations can probably be applied to the skin without causing any skin irritation.

#### 3.2.8. Encapsulation Efficiency and Assay

Encapsulation efficiency refers to the ratio of entrapped drug molecules within a formulation and the free drug molecules within a sample. It is often reported as percentage encapsulation efficiency (%EE) [77]. In this study, %EE was defined for each individual drug as the phase in which it was supposed to be dissolved. As stipulated in the methodology, all formulations that were deemed acceptable were centrifuged at 25,000 rpm (25 °C) for 45 min. Care was taken not to centrifuge at the highest speed of the apparatus so as not to “break” the emulsion droplets and subsequently release the encapsulated drug in the dispersed phase. The OLV microemulsions did not show any signs of phase separation after the centrifugation process, unlike the other formulations. No signs of physical instability were observed, and no supernatant was obtained for these formulations. Consequently, the %EE could not accurately be determined. It was, therefore, presumed that all OLV microemulsions gave 100% EE. Following, the INH and RIF concentrations in the finally prepared formulations were determined using HPLC analysis, and the results were subsequently compared. The drug concentrations are reported in Table 6.

From Table 6, it is clear that the %drug concentration overall is markedly higher in the OLV microemulsion formulations tested compared to the FRK and LEM nanoemulsions. This finding is supported by the results obtained for %EE, where it was indicated that OLV microemulsions exhibited the highest %EE. It seems as though the larger droplets could include more dissolved FDC overall, since the only differences between OLV325, FRK325, and LEM325 (except for the oil phase) are the average droplet sizes and the size distribution of these formulations. Similarly, the %RIF incorporated into the formulations is generally higher than the %INH included. Interestingly, it also appears that as the water phase of the different formulations increased and the oil phase decreased, the %INH obtained increased, whereas the %RIF decreased. Additionally, the type of oil included in the emulsions plays a significant role, as little (FRK325) to no %INH (LEM325) could be established for the different formulations. Another possible reason might be the acidity of the oil phase included. As indicated previously, RIF is known to naturally degrade in more acidic environments. The pH of OLV has been found to vary between a value of 4 and 5 and fluctuates more intensely when there is a temperature variation [97]. The literature has furthermore shown that LEM comprises a pH value of 3.5 [128], indicating that LEM is more acidic compared to OLV. Unfortunately, no pH for FRK could be established in the literature. Nevertheless, this distinction in acidity might be the reason why a difference is seen in the %RIF obtained from the different SEDDSs, as the RIF would display faster degradation in the more acidic milieu.

### 3.3. Topical Delivery of a Fixed-Dose INH-RIF Combination

After concluding characterization testing, OLV415, OLV325, and OLV316 were considered the most suitable (although not ideal) for subjection to topical drug delivery experimentation. This is because these formulations depicted the most physically stable profiles and also comprised the highest concentrations of INH and RIF in their individual microemulsion formulations compared to those of the FRK and LEM nanoemulsion formulations. Although no clear ranking order could be established between these formulations, it did seem from the results obtained that OLV325 could be considered slightly more acceptable, as this formulation included the highest concentration of INH and RIF in combination. Moreover, it could be established that the larger the oil droplets in which the FDC was dissolved, and the lower the repulsive forces between the individual droplets, the more readily INH and RIF were encapsulated in the different formulations. Additionally, the formation of microemulsions comprising OLV was able to effectively separate the drugs from each other to reduce the INH-accelerated decomposition of RIF, since detectable concentrations of RIF could still be obtained after 6 h of testing at an increased temperature (i.e., 32 °C), as seen in Table 7. Subsequently, INH and RIF release studies were first conducted where synthetic membranes, i.e., polytetrafluoroethylene membranes, were used to carry out experiments before dermal drug diffusion was analyzed.

All tested OLV microemulsions released notably measurable INH concentrations, as all of these formulations displayed an approximate amount of drug that diffused through the indicated membranes into the receptor compartments of the Franz diffusion cells. However, only OLV325 was able to release a sufficient concentration of RIF that could be quantified. The acquired data are indicated as a percentage of the initial amounts of INH and RIF incorporated into the finally selected formulations, as shown in Table 7.

From the results obtained, the average %INH released could be ranked as OLV316 >> OLV325 > OLV415, where only OLV325 released RIF in quantifiable concentrations. This microemulsion formulation comparatively contained the highest OLV ratio, which could possibly be the reason for the amount of RIF that was released by the individual microemulsions, where the formulations that comprised less OLV (OLV316 and OLV415) could not release RIF to the same extent. Interestingly, the higher the water phase concentration and the lower the surfactant phase concentration, the more INH (and RIF) was released. Normally, as the amount of the surfactant phase decreases, the surface tension between the oil phase and the aqueous phase of the formulations increases, which subsequently reduces the ability of these formulations to prevent the coagulation of the individual small droplets [44,45], which in turn causes larger droplets to form. This observation is supported by the results acquired for the average droplet size of the different microemulsion formulations, as OLV325 depicted the largest average droplet size of the OLV comprising emulsions. Furthermore, OLV325 also presented with the lowest cloud point compared to the other OLV microemulsions, which indicates that this formulation will release the included drugs faster at a lower temperature (<32 °C) relative to the other OLV microemulsions due to excipient dehydration [22,62,125].

In order to enhance RIF detection, the selected microemulsion formulations were prepared using a fairly dated method, termed supersaturation. However, this method has not yet been optimized [22,76,129]. During the preparation method, an excess amount of RIF was mixed with the OLV, followed first by slightly heating the dispersion to increase the dissolution of the drug, before the mixture was cooled to room temperature. This heating and cooling process was believed to be reasonably normal, as numerous emulsions are typically formulated in this manner. Furthermore, the delivery of RIF (and INH) in supersaturated concentrations to the skin is preferred, as these systems will then almost certainly improve the dermal drug flux [130] due to the formulations that impede nucleation and crystal growth because of the increased kinetic and thermodynamic retardation of drug precipitation. The OLV microemulsions were consequently stored at ambient temperature for 24 h prior to visual assessment for any signs of drug precipitation. As no drug precipitation was observed, the microemulsions were considered adequate for dermal diffusion.

Despite the drug release profiles obtained for the OLV formulations, INH or RIF amounts could not be quantified in any of the microemulsion receptor phases after dermal drug diffusion studies. Thus, transdermal drug delivery could not be established for any OLV microemulsions. However, these results could still prove beneficial for the treatment of CTB, as this dosage form is developed to be applied directly to skin lesions, and affected individuals are likely already receiving systemic treatment for pulmonary TB [18,19,22]. These topical microemulsions should rather address the skin condition itself. Moreover, systemic drug interactions and other side effects will probably also be avoided in this manner, while the bacilli are still targeted.

Furthermore, neither INH nor RIF could be detected in the SC–epidermis layer after tape stripping. Similarly, no drugs were detected in the epidermis–dermis layers of the skin. Poor dermal drug delivery can possibly be attributed to several factors, such as drug solubility, formulation instabilities, or drug interactions, once INH and RIF were combined in these formulations. The stability of INH and RIF has also only been determined in low-pH aqueous solvents [5,131,132,133,134], simulating the gastrointestinal environment. No studies could be found in which the stability of these drugs was analyzed in other solvents, for example, oils. Therefore, it was consequently decided to determine the stability of INH and RIF in a more alkaline liquid environment.

### 3.4. Stability Determination of INH and RIF

The degradation of INH and RIF was analyzed over a 24 h period to determine the effect of varying the temperature on the degradation of these drugs. Each prepared sample was diluted with the described solvent (Section 2.4.9), which had a final pH of 6.8. The results obtained at 5, 25, and 40 °C after 24 h are listed in Table 8, where the %drug that could be quantified is specified. From these findings, it is clear that both INH and RIF showed some degree of degradation; however, the degree of degradation of RIF was more pronounced than that of INH.

From the results listed in Table 8, it could be established that the concentration of INH decreased by approximately 12% when stored at 40 °C, while the %RIF decreased significantly when retained at higher temperatures (>5 °C) for only 24 h (12.3% and 33.4% at 25 °C and 40 °C, respectively). As a result, it could be confirmed that INH is relatively stable at lower temperatures, while the degradation of RIF is highly sensitive to temperature fluctuation, as indicated in the literature [134,135,136]. These results can clarify the findings achieved during skin diffusion studies, where the different microemulsion formulations analyzed were exposed to a temperature of 32 °C ± 0.5 °C (skin surface temperature). This increased temperature, together with the acidic environment created by OLV, most likely led to the accelerated degradation of RIF and the low concentration values detected for RIF during the analysis process. Furthermore, it is well-known that the degradation of RIF is accelerated in the presence of INH, which was also present in these formulations.

**Figure 6:**
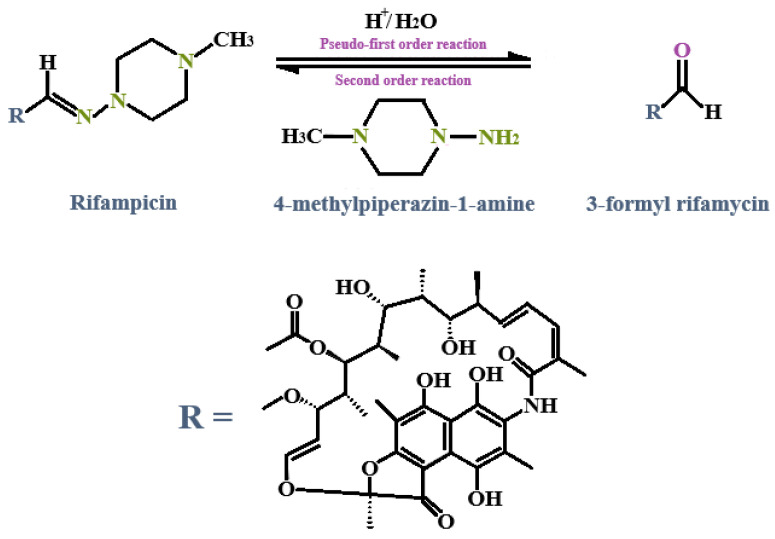
Degradation of RIF under acidic conditions due to reversible acid hydrolysis of the azomethine group, which results in the formation of 3-formyl rifamycin (3-FR) and 1-amino-4-methylpiperazine [110].

The chemical interaction that occurs between INH and RIF in an acidic solvent is a recognized challenge when it comes to formulating stable FDC dosage forms. RIF has the unique ability to exist as zwitterion, depending on the pH of the environment. The 4-hydroxy functional group has been linked to a pKa of 1.7, while a pKa value of 7.9 can be ascribed to the 3-piperazine nitrogen group [117,137]. It has been stated that the optimal pH environment for maximum RIF solubility is around 1–3. However, its solubility has not really been studied in alkaline solvents, since most formulations are administered orally, which leads to high drug solubility in the gastric environment due to the protonation of RIF, which can easily transpire in the acidic milieu of the stomach. Furthermore, what additionally complicates the formulation of RIF into an acceptable drug delivery system is the fact that the degradation of RIF (which also mostly occurs at low pH) is a complex process, as this drug can undergo many chemical reactions, including acid hydrolysis or oxidation, both of which ultimately affect the overall stability of this drug [6,110]. In other words, the degradation of RIF is both temperature- and pH-dependent. Normally, under acidic conditions, the degradation of RIF occurs due to reversible acid hydrolysis of the azomethine group, resulting in the formation of two by-products, namely, 3-formyl rifamycin (3-FR) and 1-amino-4-methylpiperazine (Figure 6) [5,94,110,133,138].

**Figure 7:**
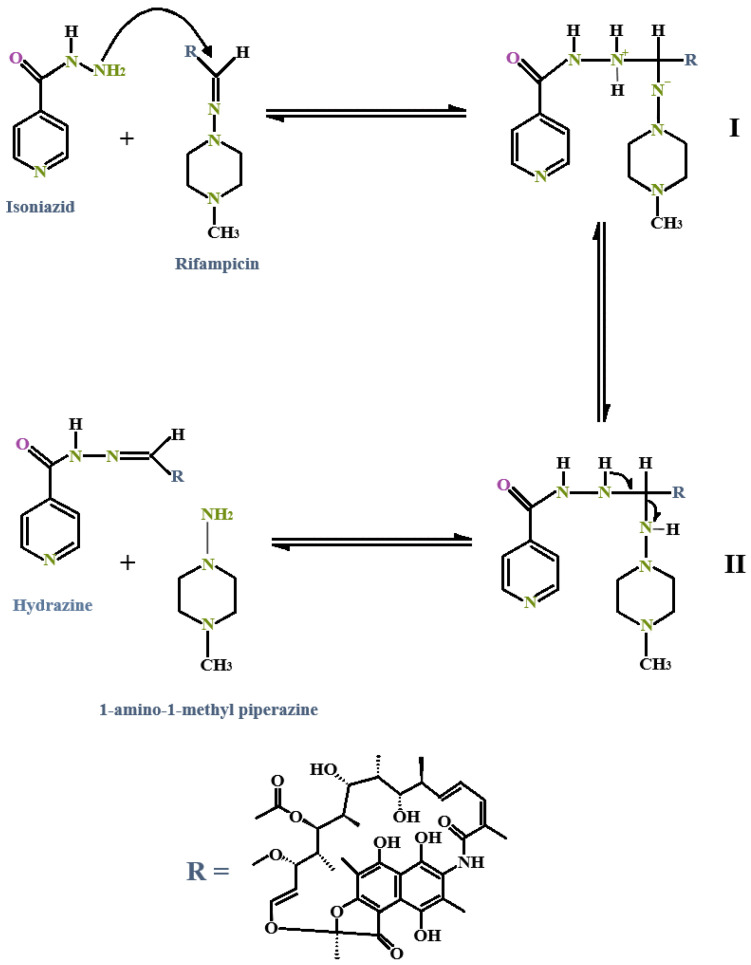
The interaction between INH and RIF with subsequent formation of isonicotinyl hydrazone (HYD) [5,9]. (**I**): Intermediate 1. (**II**): Intermediate 2.

Interestingly, this reaction follows pseudo-first-order kinetics, while the reverse reaction that occurs between the two by-products follows a second-order reaction. RIF degradation alone would normally cease after the formation of 3-FR; however, in the presence of INH, a catalytic reaction occurs where 3-FR binds irreversibly to INH and forms isonicotinyl hydrazone (HYD). This reaction occurs rapidly and has been described as following second-order kinetics [110,113,139]. This insoluble HYD metabolite further contributes to the poor bioavailability of RIF [5,6,9,115]. What is more, the presence of INH has been shown to increase the rate of RIF degradation when combined in an FDC. The interaction between INH and RIF to form HYD ensues due to a direct reaction owing to the nucleophilic attack of the amino group from the INH structure on the imine group of the RIF structure through a tetrahedral mechanism, as shown in Figure 7 [5,9,113,134]. Remarkably, the instability of HYD in acidic conditions facilitates the conversion of HYD back into INH and 3-FR, following pseudo-first-order kinetics, ultimately resulting in the regeneration of INH, while RIF is broken down and lost [110,113,139].

As mentioned, the pH of OLV can range from 4 to 5; however, temperature variation also affects the final pH of this oil, and it can thus be described as more acidic in nature. The incorporation of RIF into OLV, along with the heating procedure during the formulation process, could subsequently possibly have contributed to the increased degradation of RIF, where the formation of 3-FR transpired more rapidly. Furthermore, with the addition of INH into the water phase of the different formulations, it is possible that the formation of HYD also occurred. However, the exact degree of degradation of RIF and INH within these formulations was not tested because it fell outside the scope outlined for this study.

## 4. Conclusions

The benefits of nanocarriers, such as emulsions and SEDDSs, are numerous, including but not limited to controlled drug release, improved drug delivery, and physical protection against some physiological characteristics that could affect degradation, including pH changes [140]. Transdermal drug delivery of, for example, INH and RIF is not a novel concept concerning the formulation of effective dosage forms for the successful treatment of TB; however, their formulation in topical emulsions (nanoemulsions and microemulsions) has not been studied [54,140,141,142]. These emulsions are one of many forms of lipophilic nanocarriers that offer the potential to increase the solubility and drug delivery of lipophilic and hydrophilic drugs alike [143]. The goal of formulating topical emulsions using the self-emulsifying mechanism is to deliver the drug(s) directly to the site of action (in this study, CTB lesions) to establish a localized pharmaceutical effect. To achieve this, the drug(s) included should have the ability to cross the SC layer and diffuse into the epidermal layer of the skin [22,79]. Therefore, the careful selection of excipients and their ratios is crucial during formulation to ensure that these excipients further enhance the solubility of the selected drug(s) and simultaneously improve the stability of the overall formulation.

It was clearly noted that the solubility of RIF increased significantly when it was dissolved in FRK and LEM, whereas the solubility of INH in water correlated well with values found in the literature. INH could not be detected in any of the oil phases, confirming its highly hydrophilic nature [144]. The choice of oil not only affects the solubility of INH and RIF, but a significant difference in droplet size was also observed between the various types of oils included in the SEDDS formulations, despite using the same excipient ratios. However, smaller water ratios with higher surfactant ratios did deliver formulations with smaller droplets; moreover, these formulations were unable to effectively deliver the drugs through the skin compared to emulsions with larger dispersed droplets. Furthermore, a correlation between zeta potential and droplet size was observed, where a decrease in zeta potential was evident as the average droplet size increased, ultimately resulting in less stable formulations according to the criteria stipulated. Also, prolonged self-emulsification times can be seen as an indication of the occlusivity of emulsions, which could, in turn, improve drug delivery as a result [145]. Consequently, it could be concluded that the average droplet size of an emulsion affects various physical characteristics of these formulations. Theoretically, the FRK and LEM emulsion formulations displayed a general improvement in the physical characteristics compared to the OLV emulsions; however, the instabilities observed with these essential oils resulted in their ultimate exclusion from this study. It is important to reiterate that droplet size was only visually analyzed with a ZEISS LSM 980 confocal laser scanning microscope, and not all measurements could be accurately attained for the different formulations. The individual droplets were possibly too small to measure accurately using this microscope. Furthermore, because of the placement of microscope slides on the emulsions, the droplets were probably compressed, which skewed the dimensions, creating the impression of larger droplets. It is therefore suggested that orthogonal techniques, such as scanning electron microscopy, transmission electron microscopy, and/or using a Zetasizer, be employed in future studies to measure the droplet sizes of these types of topical drug delivery systems more precisely, in order to also predict formulation stability more accurately.

Furthermore, although the OLV formulations portrayed drug release during the 6 h membrane diffusion studies, low drug permeation was observed during the skin diffusion studies executed. A contributing factor was probably the less-than-ideal physical characteristics, which likely affected the permeability of these emulsions through the skin. In fact, the elevated temperatures at which these formulations were formulated, as well as the acidic environments that transpired as a result of the oils utilized, greatly affected the decomposition of INH and RIF.

Emulsions for the topical administration of INH and RIF prepared by the self-emulsification mechanism have great potential for the treatment of CTB; however, careful consideration of the types of oil phase and surfactants, as well as their ratios, should be given prior to inclusion into topical formulations to ensure that these excipients do not negatively interact with the included drugs. Furthermore, the manufacturing technique should be taken into consideration. One method to decrease the droplet size is sonication. This method is still considered a low-energy technique compared to its high-energy approaches, i.e., high-pressure homogenizers utilized in the formulation of nanoemulsions [146,147]. Additionally, the inclusion of antioxidants, for example, α-tocopherol, which is not only lipid-soluble with numerous beneficial effects on the skin but also assists in preventing the decomposition of fatty acids in an oil [148].

The focus of current research has shifted to improving existing therapies and refining drug delivery. Topical and/or transdermal routes offer noninvasive methods that mostly avoid first-pass metabolism, which subsequently leads to a decrease in the side effects experienced through other drug delivery routes [55]. Although many challenges are associated with this administration route, the formulation of targeted transdermal drug delivery systems could ultimately be used to deliver drugs more effectively by also using the lymphatic system, which has many potential benefits, especially in the treatment of diseases such as endogenous EPTB, parasitic infections, metastatic cancers, and human immunodeficiency virus [22,55,149,150,151].

## Figures and Tables

**Table 1:** Gradient program used for HPLC analysis.

Time (min)	Mobile Phase A	Mobile Phase B	Elution
0	100%	0%	Equilibration
0–5	100%	0%	Isocratic
5–6	100→0%	0→100%	Gradient
6–15	0%	100%	Isocratic
15–20	0→100%	100-0%	Gradient
20–25	100%	0%	Isocratic

**Table 2:** Summary of validation parameters and results obtained.

Parameter	Isoniazid	Rifampicin
**Specificity**	No interference by oils, surfactants, or solvents was detected	No interference by oils, surfactants, or solvents was detected
**Range of the analytical method**	16–100 µg/mL	32–200 µg/mL
**Linearity**	r^2^ = 0.9953	r^2^ = 0.9973
**Accuracy:** % Recovery at the specified concentrations	**~16 µg/mL: 99.4%** (%RSD_(n=3)_: 0.3%) **~40 µg/mL: 100.4%** (%RSD_(n=3)_: 0.2%) **~100 µg/mL: 100.0%** (%RSD_(n=3)_: 0.0%)	**~32 µg/mL: 102.7%** (%RSD_(n=3)_: 0.4%) **~80 µg/mL: 98.5%** (%RSD_(n=3)_: 0.3%) **~200 µg/mL: 100.2%** (%RSD_(n=3)_: 0.6%)
**Precision:** Repeatability at ~40 µg/mL for INH and ~80 µg/mL for RIF (n = 6)	0.2%	0.4%
Intermediate precision at the specified concentrations	**~16 µg/mL:** %RSD_(n=3)_: 0.3% **~40 µg/mL:** %RSD_(n=3)_: 0.2% **~100 µg/mL:** %RSD_(n=3)_: 0.0%	**~32 µg/mL:** %RSD_(n=3)_: 0.4% **~80 µg/mL:** %RSD_(n=3)_: 0.3% **~200 µg/mL:** %RSD_(n=3)_: 0.6%

**Table 3:** A list of the abbreviations and ratios of the checkpoint SEDDS formulations developed.

Oil Phase	Formulation Abbreviation	Excipient Ratios *Surfactant Phase:Oil Phase:Aqueous Phase*
Olive oil Frankincense oil Lemon oil	OLV442 FRK442 LEM442	4:4:2
Olive oil Frankincense oil Lemon oil	OLV352 FRK352 LEM352	3:5:2
Olive oil Frankincense oil Lemon oil	OLV343 FRK343 LEM343	3:4:3
Olive oil Frankincense oil Lemon oil	OLV415 FRK415 LEM415	4:1:5
Olive oil Frankincense oil Lemon oil	OLV325 FRK325 LEM325	3:2:5
Olive oil Frankincense oil Lemon oil	OLV316 FRK316 LEM316	3:1:6

**Table 4:** The emulsification classification system typically used for SEDDSs behavior upon dilution.

Emulsification Grading	Time to Self-Emulsify	Description
A	Under 1 min	Emulsion displays quick emulsification with a bluish/clear appearance
B	Under 1 min	Emulsion forms rapidly with a bluish appearance
C	Within 2 min	Emulsion displays a milky appearance with very fine droplets
D	Over 2 min	Emulsion undergoes slow emulsification, with a dull, greyish appearance in addition to the formation of oily droplets
E	Over 2 min	Emulsion presents with large oil droplets on the surface, with poor emulsification ability

**Table 5:** Characterization results obtained for topical formulations that did not show any phase separation, flocculation, or drug precipitation after storage for 24 h at an ambient temperature. Results that did not meet the set criteria for a particular test are highlighted, and values are indicated in bold.

Formulations	Zeta Potential (mV)	Droplet Size (μm)	PDI	pH	Self-Emulsification Time (min)	Self-Emulsification Grading	Viscosity (mPa.s)	Cloud Point (°C)
**OLV343**	–24.00	*	0.36	7.00	18.33	D	47 820.67	**27.6**
**OLV415**	–31.47	4.876	**0.74**	6.95	29.14	D	45 132.30	**27.7**
**OLV325**	–31.73	4.839	**1.00**	6.97	6.58	D	17 863.43	**26.3**
**OLV316**	–27.57	6.596	**0.97**	7.05	11.56	D	17 418.28	**28.0**
**FRK415**	–41.00	*	0.43	6.99	60.33	E	56 763.67	39.4
**FRK325**	–25.83	*	0.47	7.10	60.33	E	65 674.00	51.5
**FRK316**	–30.77	5.056	0.56	7.10	59.21	E	45 167.33	**31.1**
**LEM415**	–29.00	*	0.58	6.80	63.40	E	52 547.00	45.5
**LEM325**	–37.07	7.676	0.46	6.88	63.40	E	7 331.60	43.0
**LEM316**	–37.07	6.028	0.53	6.96	63.40	E	18 816.70	42.9

* = Droplets were too small to accurately measure the sizes of the individual droplets by means of the ZEISS LSM 980 confocal laser scanning microscope.

**Table 6:** Percentage INH and RIF concentrations of the selected formulations. Values are presented as the mean ± standard deviation.

%INH	Formulations	%RIF
68.2 ± 0.5 91.6 ± 0.7 85.1 ± 0.2 90.4 ± 0.9	**OLV343** **OLV325** **OLV415** **OLV316**	111.5 ± 2.1 109.5 ± 1.0 109.1 ± 1.3 107.6 ± 0.5
57.4 ± 0.4	**FRK325**	83.3 ± 3.9
–	**LEM325**	87.6 ± 0.1

**Table 7:** Results for membrane release experiments conducted over 6 h. The average cumulative concentrations as well as the average percentage of drug released are shown as the mean ± standard deviation.

INH					RIF	
% INH Released	Release Rate (μg.cm^2^/h)	Cumulative Amount (μg/cm^2^)		% RIF Released	Release Rate (μg.cm^2^/h)	Cumulative Amount (μg/cm^2^)
7.68 (±2.22)	9.49	59.93 ± 0.99	**OLV415**	–	–	–
15.00 (±0.89)	16.48	117.07 ± 0.99	**OLV325**	3.32 (±0.46)	3.78	31.21 (±3.66)
24.07 (±1.51)	26.60	176.39 ± 0.99	**OLV316**	–	–	–

**Table 8:** Percentage of intact INH and RIF detected after 24 h exposure to various temperatures.

Temperature (°C)	INH Concentration (μg/mL)	%INH	RIF Concentration (μg/mL)	%RIF
**5**	81.69	99.9	156.80	96.8
**25**	76.56	97.3	142.06	87.7
**40**	71.79	87.8	76.56	66.6

## Data Availability

The raw data supporting the conclusions of this article will be made available by the authors upon request.

## Abbreviations

The following abbreviations are used in this manuscript:

%EE	Encapsulation efficiency
3-FR	3-Formyl rifamycin
CTB	Cutaneous tuberculosis
EPTB	Extrapulmonary tuberculosis
FDCs	Fixed-dose combinations
FRK	Frankincense oil
HYD	Isonicotinyl hydrazone
INH	Isoniazid
LEM	Lemon oil
o/w	Oil-in-water
OLV	Olive oil
PBS	Phosphate buffer solutions
PDI	Polydispersity index
PTB	Pulmonary tuberculosis
RBF	Rose blend fragrance
RIF	Rifampicin
SC	Stratum corneum
SEDDSs	Self-emulsifying drug delivery systems
STA	Simultaneous thermal analysis
TAM	Thermal activity monitor
TB	Tuberculosis
TTO	Tea tree oil
w/o	Water-in-oil
